# LncRNA MCF2L-AS1 aggravates the malignant development of colorectal cancer via targeting miR-105-5p/RAB22A axis

**DOI:** 10.1186/s12885-021-08668-w

**Published:** 2021-09-30

**Authors:** Wencheng Kong, Hui Li, Lesi Xie, Guangxing Cui, Weigang Gu, Hongchen Zhang, Wencong Ma, Yifeng Zhou

**Affiliations:** 1 Department of Gastrointestinal Surgery, Affiliated Hangzhou First People’s Hospital, Zhejiang University School of Medicine, Hangzhou, 310006 Zhejiang China; 2 Department of Gastroenterology, Affiliated Hangzhou First People’s Hospital, Zhejiang University School of Medicine, No. 261 Huanshan Road, Hangzhou, 310006 Zhejiang China; 3 Department of Pathology, Affiliated Hangzhou First People’s Hospital, Zhejiang University School of Medicine, Hangzhou, 310006 Zhejiang China

**Keywords:** MCF2L-AS1, miR-105-5p, RAB22A, Colorectal cancer

## Abstract

**Background:**

Colorectal cancer (CRC) represents one of the major malignant cancers in the world. It has been demonstrated that long non-coding RNAs (lncRNAs) can cause great influences on various human cancers. Though MCF.2 cell line derived transforming sequence like antisense RNA 1 (MCF2L-AS1) and its carcinogenic effect in CRC has been elucidated by several previous researches, the underlying mechanism remains unknown.

**Aim:**

We aimed at exploring the function and regulatory mechanism of MCF2L-AS1 in CRC.

**Methods:**

MCF2L-AS1 expression in CRC cells was tested via RT-qPCR assay. The effects of MCF2L-AS1 on the biological properties of CRC cells were testified through functional experiments. The molecular mechanism of MCF2L-AS1 was verified through mechanism experiments.

**Results:**

MCF2L-AS1 was highly expressed in CRC cells, and it could enhance the proliferation, migration, invasion and epithelial-mesenchymal transition (EMT) process of CRC cells. MiR-105-5p was sponged by MCF2L-AS1 in CRC cells and Ras-related protein Rab-22A (RAB22A) was verified to be the downstream target of miR-105-5p. It was verified through rescue assays that RAB22A overexpression or miR-105-5p silencing could reverse the repressive impact of MCF2L-AS1 silencing on CRC progression.

**Conclusion:**

MCF2L-AS1 accelerated the malignant development of CRC cells by targeting the miR-105-5p/RAB22A axis.

**Supplementary Information:**

The online version contains supplementary material available at 10.1186/s12885-021-08668-w.

## Background

As one of the commonest gastrointestinal cancers worldwide, colorectal cancer (CRC) is featured by high incidence and mortality. Even if the treatment strategies for CRC have been constantly optimized, the overall survival of CRC patients remains relatively low [1]. Therefore, it is necessary to find more biomarkers so as to provide effective therapeutic options for CRC treatment.

LncRNAs are defined as RNA molecules with over 200 nucleotides and without protein coding capacities [2, 3]. It has been proven that lncRNAs are capable of regulating gene expression, genome activity, histone modifications, DNA methylation, chromatin remodeling, etc. [4–6]. In addition, lncRNA expression has been proved to be negatively related to DNA methylation during the onset of puberty [7]. The interaction between lncRNAs and chromatin-modifying enzymes can control chromatin structure [8]. Lately, some lncRNAs have been reported to participate in regulating cancer development, which includes CRC [9–11]. For instance, Liu et al. have disclosed that 79 lncRNAs are closely related to the early-stage CRC such as ELFN1-AS1, LINC01234, SNHG17, etc. That finding lays the ground for the treatment for CRC patients and has potential diagnostic value [12]. Besides, it has also been confirmed that XXbac-B476C20.9 is closely associated with the prognosis of CRC, which may affect the overall survival of CRC patients [13].

As a relatively newly found lncRNA, MCF2L-AS1 as well as its influence on cancer progression has not been widely studied and reported before. When we were searching for references on the relationship between MCF2L-AS1 and CRC, only two documents illustrating that MCF2L-AS1 can serve as a tumor promoter were found by us [14, 15]. Therefore, we decided to unveil the underlying mechanism of MCF2L-AS1 in CRC development, hoping to explore its potential regulatory pattern.

The fact that lncRNAs can bind to microRNAs (miRNAs) as competing endogenous RNAs (ceRNAs) and thus mediating the downstream gene expression has been disclosed by many studies [16]. In addition, ceRNA mechanism has been confirmed to perform important functions in cancer progression, including CRC. For example, lncRNA UICLM aggravates CRC liver metastasis by functioning as a ceRNA of miRNA-215 to regulate ZEB2 expression [17]. LINC00858 has been verified to aggravate CRC progression by competitively binding to miR-22-3p in CRC [18]. MFI2-AS1 has been identified to be a novel lncRNA for the prognosis of stage III/IV CRC [19]. Therefore, in our study, we also explored the ceRNA pattern of MCF2L-AS1 in CRC, trying to identify the interaction among MCF2L-AS1, miR-105-5p and RAB22A and verify their effects on CRC progression. In a word, we aimed to study the function of MCF2L-AS1 as well as its regulatory mechanism in CRC progression, hoping to offer novel sights for CRC treatment.

## Methods

### Cell culture

Four human CRC cell lines (HCT15, SW620, SW116 and LOVO) were purchased respectively from ATCC (Manassas, VA, USA). Human colon epithelial NCM-460 cell line was procured from Lonza Group Ltd. (Basel, Switzerland) and cultured in DMEM (Corning, Tewksbury, MA, USA). Among the CRC cells, LOVO cells were cultivated in F-12 K Medium; HCT15 cells were incubated in RPMI-1640 Medium, while SW116 and SW620 cells were cultivated in Leibovitz’s L-15 Medium. 10% fetal bovine serum (FBS; Gibco, Gaithersburg, MD, USA), together with 1% penicillin-streptomycin (Gibco) were used for cell culture in 5% CO_2_ at 37 °*C. Medium* was replaced every 3 days.

### Cell transfection

For the knockdown of MCF2L-AS1 and RAB22A, specific short hairpin RNAs (shRNAs) targeting MCF2L-AS1 (sh-MCF2L-AS1#1/2) and RAB22A (sh-RAB22A#1/2) were provided by GenePharma (Shanghai, China). Non-specific shRNAs were used as negative control (NC). To overexpress RAB22A, the whole sequence of RAB22A was sub-cloned into pcDNA3.1 vector (Invitrogen, Carlsbad, CA, USA) with empty vectors being utilized as NC. MiR-105-5p mimics, miR-105-5p inhibitor and NCs were purchased from Ribobio (Guangzhou, China). All transfections were carried out for 48 h with Lipofectamine 3000 (Invitrogen) according to the user’s guideline. Related plasmids quantities and transfection concentration are listed in Table 1.

### Total RNA extraction and quantitative real-time polymerase chain reaction (RT-qPCR)

In line with the instruction of TRIzol reagent (Invitrogen), total RNA was extracted in HCT15 and SW116 cells. Synthesis of complementary DNA (cDNA) was achieved with the help of PrimeScript™ II Reverse Transcriptase (TaKaRa, Shiga, Japan). RT-qPCR was implemented on ABI Prism 7900HT sequence detector (Applied Biosystems, Foster City, CA, USA) using SYBR Green I kit (TaKaRa) followed by 2^-ΔΔCt^ method. In relevant assays, GAPDH and U6 were loading controls.

### Immunofluorescence (IF) staining assay

After being washed in PBS, transfected HCT15 and SW116 cells in culture slides were fixed with 4% paraformaldehyde and blocked for 10 min with 5% BSA. The primary antibodies against Ki-67 and E-cadherin were used to be incubated with cells overnight. After being washed in PBS for three times, the slides were incubated with the secondary antibodies for 2 h. The slides were then stained using DAPI and examined using an Olympus confocal imaging system (Olympus, Tokyo, Japan). The resolution of the figures is 300 dpi and no downstream processing or averaging methods were adopted to enhance the resolution.

### 5-ethynyl-20-deoxyuridine (EdU) incorporation assay

Cells at logarithmic growth stage were taken and seeded in 96-well plates with 1 × 10^4^ cells per well to the normal growth stage. An appropriate amount of 50 μM EdU culture medium was prepared by dilution of EdU solution by 1:1000. Each well was added with 100 μL 50 μM EdU assay kit for cultivation for 2 h, and then the culture medium was discarded. DAPI was added to stain nucleus for 5 min at room temperature, and the stained cells were imaged with a fluorescence microscope (Olympus). Cell proliferation ability was assessed via the Cell-light™ EdU ApolloR567 in vitro Imaging Kit (Ribobio) according to the user guide. The resolution of the figures is 300 dpi and no downstream processing or averaging methods were adopted to enhance the resolution.

### Transwell assay

Cell migration assay was performed using Transwell chambers (24-well; Corning, Corning, NY, USA). The lower chamber was added with complete culture medium. Cells were suspended in serum-free medium and plated into the upper chamber. Then the chambers were cultivated with 5% CO_2_ at 37 °C for 24 h. Cells in the upper layer were removed with caution by a cotton swab and then the cells in the lower chamber were fixed in methanol solution for 15 min. After that, migrated cells were counted using 0.1% crystal violet dye and observed via a light microscope (DMi1, Leica, Wetzlar, Germany). Transwell chambers were pre-coated with Matrigel (Clontech, Madison, WI, USA) for invasion assay. Cell migration and invasion were determined by counting 5 random fields under a microscope (DMi1, Leica, Wetzlar, Germany).

### Flow cytometry assay

Cell apoptosis of HCT15 and SW116 was monitored by using Annexin V-FITC/PI staining kit (Invitrogen), as guided by supplier. 1 × 10^6^ cells were prepared in 6-well plates for 15 min of double-staining in the dark room. Results were examined by a flow cytometer (BD Biosciences, Franklin Lakes, NJ, USA).

### Caspase-3/8/9 activity analysis

The activity of caspase-3/8/9 was detected via the caspase-3/8/9 activity kit (Beyotime Institute of Biotechnology, Shanghai, China). The total protein of cells was obtained through lysis buffer and seeded into 96-well plates. After the incubation with reaction buffer and caspase substrate, caspase-3/8/9 activities in CRC cells was assessed by a microplate reader (51119180ET, Thermo Fisher Scientific, Rockford, IL, USA) at 405 nm.

### Fluorescence in situ hybridization (FISH) assay

The RNA FISH probe mix for MCF2L-AS1 was synthesized and produced by RiboBio. The fixed cells were treated by pepsin and dehydrated by using ethanol, followed by the incubation with probe in hybridization buffer. Nuclei were counterstained with DAPI and Olympus fluorescence microscope (DMI8, Leica, Wetzlar, Germany) was utilized to observe and analyze the stained cells. The resolution of the figures is 300 dpi and no downstream processing or averaging methods were adopted to enhance the resolution.

### Subcellular fractionation assay

Cytoplasmic and nuclear RNAs of HCT15 and SW116 cells were centrifuged and purified through a Cytoplasmic and Nuclear RNA Purification Kit (Norgen, Thorold, ON, Canada). The subcellular localization of MCF2L-AS1 in HCT15 and SW116 cells was identified via RT-qPCR assay, with GAPDH and U6 as the cytoplasm and the nucleus control, respectively. The resolution of the figures is 300 dpi and no downstream processing or averaging methods were adopted to enhance the resolution.

### RNA immunoprecipitation (RIP) assay

RIP assay was performed using the Thermo Fisher RIP kit (Thermo Fisher Scientific, Waltham, MA, USA). The collected HCT15 and SW116 cells were lysed by RIP lysis and then the cell lysates were collected. After that, magnetic beads conjugated with human Ago2 antibody or IgG antibody (as NC) were added into cell lysates and incubated overnight at 4 °C. After the RNA was purified, the enrichment of precipitated RNAs was tested by RT-qPCR assay.

### RNA pull down assay

The miR-105-5p sequences containing the wild-type (WT) and mutated (Mut) binding sites of MCF2L-AS1 or RAB22A were biotin-labeled for RNA pull down assay. The acquired probes were incubated with protein extracts as well as magnetic beads with Bio-NC as the control. Finally, the precipitated proteins were eluted and analyzed using RT-qPCR assay.

### Luciferase reporter assay

The wild-type and mutant binding sites of miR-105-5p in MCF2L-AS1 fragment or RAB22A 3’UTR were sub-cloned into pmirGLO dual-luciferase vector to construct MCF2L-AS1-WT/Mut or RAB22A-WT/Mut. The pmirGLO plasmids were co-transfected with miR-105-5p mimics or NC mimics into HCT15 and SW116 cells, respectively. Finally, after 48-h transfection, cells were extracted and the luciferase activity was tested by Luciferase Reporter Assay System (Promega, Madison, WI, USA).

### Statistical analysis

All experimental procedures in this study were conducted independently for at least three times, and experimental results were exhibited as mean ± standard deviation (SD). Statistical analysis was performed using GraphPad Prism 7.0 software (Graph Pad, La Jolla, CA, USA). Data analysis between two or more groups was conducted via Student’s *t*-test or one-way analysis of variance (ANOVA). Statistics with a *p* value below 0.05 were considered to be statistically significant.

## Results

### MCF2L-AS1 is highly expressed in CRC cells and accelerates the progression of CRC

**Fig. 1:**
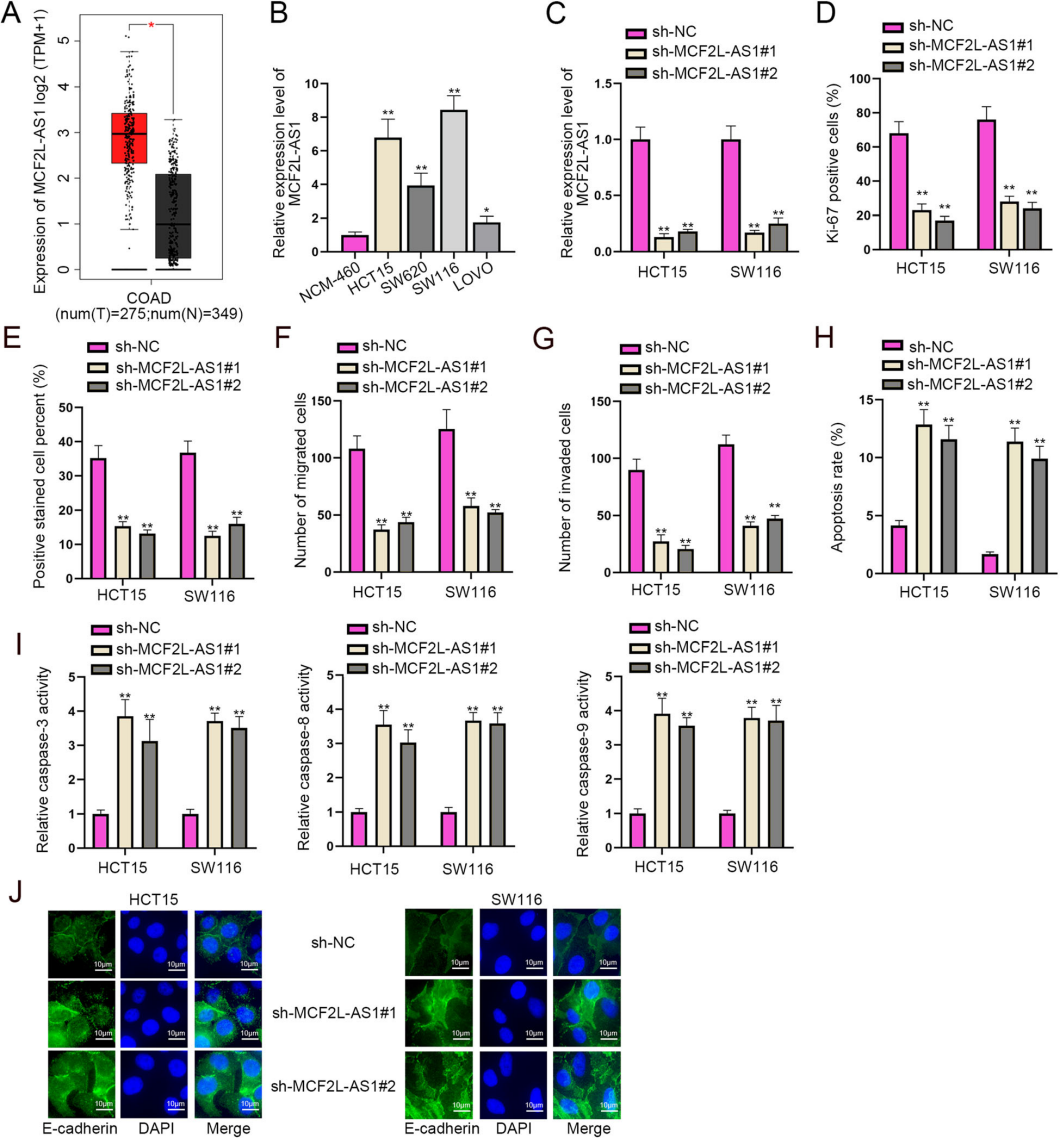
MCF2L-AS1 is highly expressed in CRC cells and accelerates the progression of CRC. **A**. The expression of MCF2L-AS1 in COAD tissues and adjacent normal tissues was assessed via GEPIA database. **B**. MCF2L-AS1 expression was measured in CRC cell lines (HCT15, SW620, SW116 and LOVO) and human normal colon epithelial cells (NCM-460) by RT-qPCR. **C**. The interference efficiency of MCF2L-AS1 in HCT15 and SW116 cells was tested by RT-qPCR assay. **D** and **E**. Cell proliferation in HCT15 and SW116 cells after MCF2L-AS1 silencing was evaluated through IF staining and EdU assay. **F** and **G**. The migratory and invasive abilities of CRC cells upon MCF2L-AS1 silencing were evaluated through Transwell assays. **H** and **I**. Flow cytometry assay and caspase-3/8/9 activity analysis were utilized to test cell apoptosis upon MCF2L-AS1 silencing. **J**. The EMT process was assessed through IF assay after MCF2L-AS1 was silenced in HCT15 and SW116 cells. Adjustments of individual color channels were made on ‘Merge’ figures. The statistical analysis of Fig. 1B-I was tested with one-way ANOVA. *P < 0.05, **P < 0.01

First, we utilized GEPIA (http://gepia2.cancer-pku.cn/#index) to assess the expression of MCF2L-AS1 in CRC tissues and normal tissues. As shown in Fig. 1A, MCF2L-AS1 was with high expression in colon adenocarcinoma (COAD) tissues compared with that in the adjacent normal tissues. Then, we performed RT-qPCR assay to examine the expression of MCF2L-AS1 in CRC cells (HCT15, SW620, SW116 and LOVO) and human normal colon epithelial cells (NCM-460), which turned out that MCF2L-AS1 was highly-expressed in CRC cell lines in contrast with that in NCM-460 cell line (Fig. 1B). The above data suggested that MCF2L-AS1 might regulate the progression of CRC. To verify this hypothesis, we selected HCT15 and SW116 cells which had a relatively higher MCF2L-AS1 expression for further studies and then transfected them with sh-MCF2L-AS1#1 and sh-MCF2L-AS1#2. After that, RT-qPCR analysis was conducted to detect the interference efficiency of sh-MCF2L-AS1#1–2. According to the result, the expression level of MCF2L-AS1 was sharply reduced in sh-MCF2L-AS1 groups compared to sh-NC group (Fig. 1C). Next, several functional assays were respectively carried out in HCT15 and SW116 cells to further verify the influence of MCF2L-AS1 silencing on the biological properties of CRC cells. Results from IF and EdU assays revealed that the Ki-67 positive cells and EdU stained positive cells were obviously decreased by sh-MCF2L-AS1#1 and sh-MCF2L-AS1#2 compared with those in sh-NC groups, which meant that MCF2L-AS1 silencing repressed cell proliferation in CRC (Fig. 1D-E & Supplementary Fig. 1). After that, cell migration and invasion change upon MCF2L-AS1 silencing was assessed by Transwell assay, and the results demonstrated that the number of migrated and invaded cells was remarkably reduced after MCF2L-AS1 was knocked down in HCT15 and SW116 cells (Fig. 1F-G & Supplementary Fig. 1). The above data demonstrated that MCF2L-AS1 silencing repressed CRC cell migration as well as invasion. Besides, as shown by the results of flow cytometry assay, after the knockdown of MCF2L-AS1, the apoptosis of HCT15 and SW116 cells were prominently enhanced (Fig. 1H & Supplementary Fig. 1). As caspase-3, caspase-8 and caspase-9 served as critical protease associated with cell apoptosis, we conducted caspase-3/8/9 activity analysis to examine the apoptosis of CRC cells upon MCF2L-AS1 silencing. Results showed that the relative activity of caspase-3, caspase-8 and caspase-9 in those cell lines was significantly enhanced upon MCF2L-AS1 silencing. The above data indicated that MCF2L-AS1 silencing promoted cell apoptosis of CRC (Fig. 1I). Besides, it was shown from IF assay that the level of E-cadherin (the epithelial marker) was prominently elevated by MCF2L-AS1 knockdown (Fig. 1J), which indicated that MCF2L-AS1 knockdown repressed the EMT process of CRC cells. Collectively, MCF2L-AS1 accelerates cell proliferation, migration, invasion and EMT process while reducing cell apoptosis in CRC.

### MiR-105-5p is sponged by MCF2L-AS1 and acts as a tumor-suppressor in CRC

**Fig. 2:**
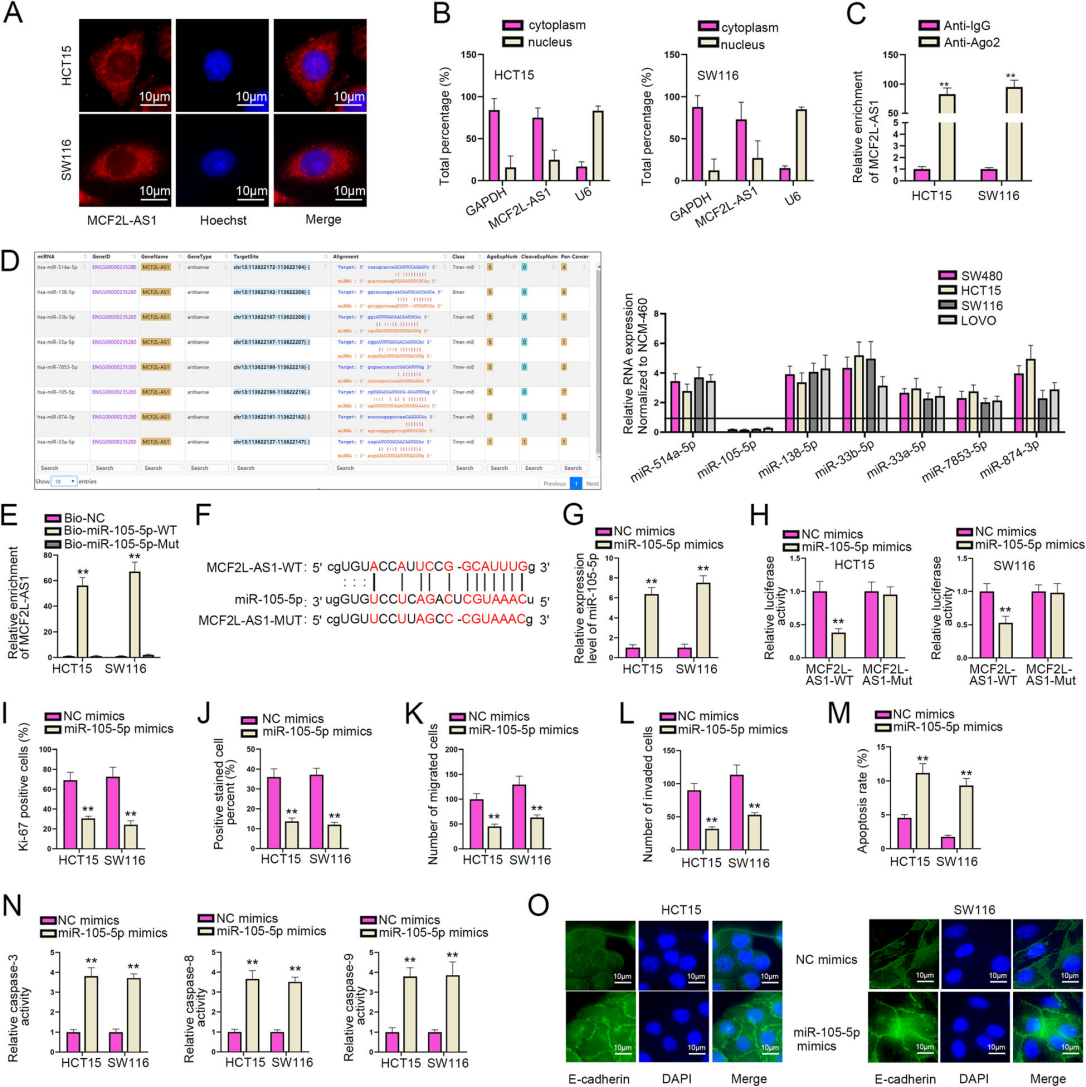
MiR-105-5p is sponged by MCF2L-AS1 and acts as a tumor-suppressor gene in CRC. **A** and **B**. The location of MCF2L-AS1 in CRC cells was assessed by subcellular fractionation and FISH assays. **C**. The enrichment of MCF2L-AS1 in RISC was tested via RIP assay. **D**. The potential miRNAs that could bind to MCF2L-AS1 was displayed (left). RT-qPCR assay was used to test the expression of seven potential miRNAs that could bind to MCF2L-AS1 (right). **E**. The binding capacity between MCF2L-AS1 and miR-105-5p was verified through RNA pull down assay. **F**. The binding sites between MCF2L-AS1 and miR-105-5p were predicted by ENCORI website. **G**. The overexpression efficiency of miR-105-5p was examined by RT-qPCR assay. **H**. Luciferase reporter assay was carried out to testify the combination between MCF2L-AS1 and miR-105-5p. **I** and **J**. Cell proliferation effected by miR-105-5p overexpression was detected by IF and EdU assays. **K** and **L**. Cell migration and invasion upon miR-105-5p overexpression were evaluated by Transwell assay. **M** and **N**. Cell apoptosis was assessed by flow cytometry assay and caspase-3/8/9 activity analysis. **O**. The EMT process upon miR-105-5p overexpression was measured through IF assay. Adjustments of individual color channels were made on ‘Merge’ figures. The statistical analysis of Fig. 2H was tested with two-way ANOVA, and the statistical analysis of Fig. 2B-E, G, I-N was tested with one-way ANOVA. **P < 0.01

To further explore the regulatory mechanism of MCF2L-AS1 in CRC, we decided to firstly find the potential target of MCF2L-AS1. Firstly, subcellular fractionation and FISH assays were conducted to identify the localization of MCF2L-AS1 in CRC cells. The findings suggested that MCF2L-AS1 mainly existed in the cytoplasm, indicating that it might regulate the expression of its downstream gene at the post-transcriptional level (Fig. 2A-B). Next, RIP assay was carried out by us and the result revealed that MCF2L-AS1 in HCT15 and SW116 cells was enriched in Anti-Ago2 group rather than in Anti-IgG group (Fig. 2C). Those findings showed that MCF2L-AS1 possibly served as a ceRNA in CRC cells to regulate its downstream gene. Next, we continued our work in determining the underlying target miRNAs that could combine with MCF2L-AS1. With the application of ENCORI (http://starbase.sysu.edu.cn/) database, potential miRNAs of MCF2L-AS1 were predicted. As a result, seven miRNAs were sifted out (miR-514a-5p, miR-105-5p, miR-138-5p, miR-33b-5p, miR-33a-5p, miR-7853-5p and miR-874-3p) (Fig. 2D, left). RT-qPCR assay was then adopted to examine the expression of the above miRNAs in CRC cells. As displayed in Fig. 2D (right), only miR-105-5p was obviously down-regulated in CRC cells compared with the other miRNAs (normalized to that in NCM-460 cell line). Therefore, miR-105-5p was kept for the following assays. To further verify the relationship between miR-105-5p and MCF2L-AS1, RNA pull down assay was conducted. It was exhibited that MCF2L-AS1 was enriched in the Bio-miR-105-5p-WT group instead of Bio-miR-105-5p-Mut (Fig. 2E), which indicated the binding capacity between MCF2L-AS1 and miR-105-5p. Next, the binding sites between MCF2L-AS1 and miR-105-5p were predicted through ENCORI website and presented in Fig. 2F. After that, we transfected HCT15 and SW116 cells with miR-105-5p mimics and RT-qPCR assay was utilized to evaluate the overexpression efficiency of miR-105-5p (Fig. 2G). Luciferase report assay was then carried out to detect the luciferase activity under different conditions, and results showed that after the transfection of miR-105-5p mimics, the luciferase activity of MCF2L-AS1-WT was declined in HCT15 and SW116 cells, while the luciferase activity of MCF2L-AS1-Mut displayed no obvious change (Fig. 2H). Considering the results above, miR-105-5p was regarded as a possible target of MCF2L-AS1 in the progression of CRC. Therefore, to explore the role and function of miR-105-5p was viewed as necessary. The results of IF and EdU assays illustrated that miR-105-5p up-regulation could effectively suppressed CRC cell proliferation in comparison with NC groups (Fig. 2I-J & Supplementary Fig. 1). Similarly, the outcome of Transwell assay also demonstrated that increased miR-105-5p expression apparently restrained the migratory and invasive abilities of CRC cells (Fig. 2K-L & Supplementary Fig. 1). In addition, it was observed from flow cytometry and caspase-3/8/9 activity analysis that the transfection of miR-105-5p mimics could significantly enhance the apoptosis of CRC cells compared with NC mimics group (Fig. 2M-N & Supplementary Fig. 1). Additionally, it was shown from IF assay that miR-105-5p overexpression resulted in an elevated level of E-cadherin, indicating that miR-105-5p overexpression also inhibited the EMT process of CRC cells (Fig. 2O). Taken together, miR-105-5p is capable of being sponged by MCF2L-AS1 and plays a tumor-suppressing role in CRC progression.

### Knockdown of miR-105-5p reverses the effects of MCF2L-AS1 down-regulation on CRC progression

**Fig. 3:**
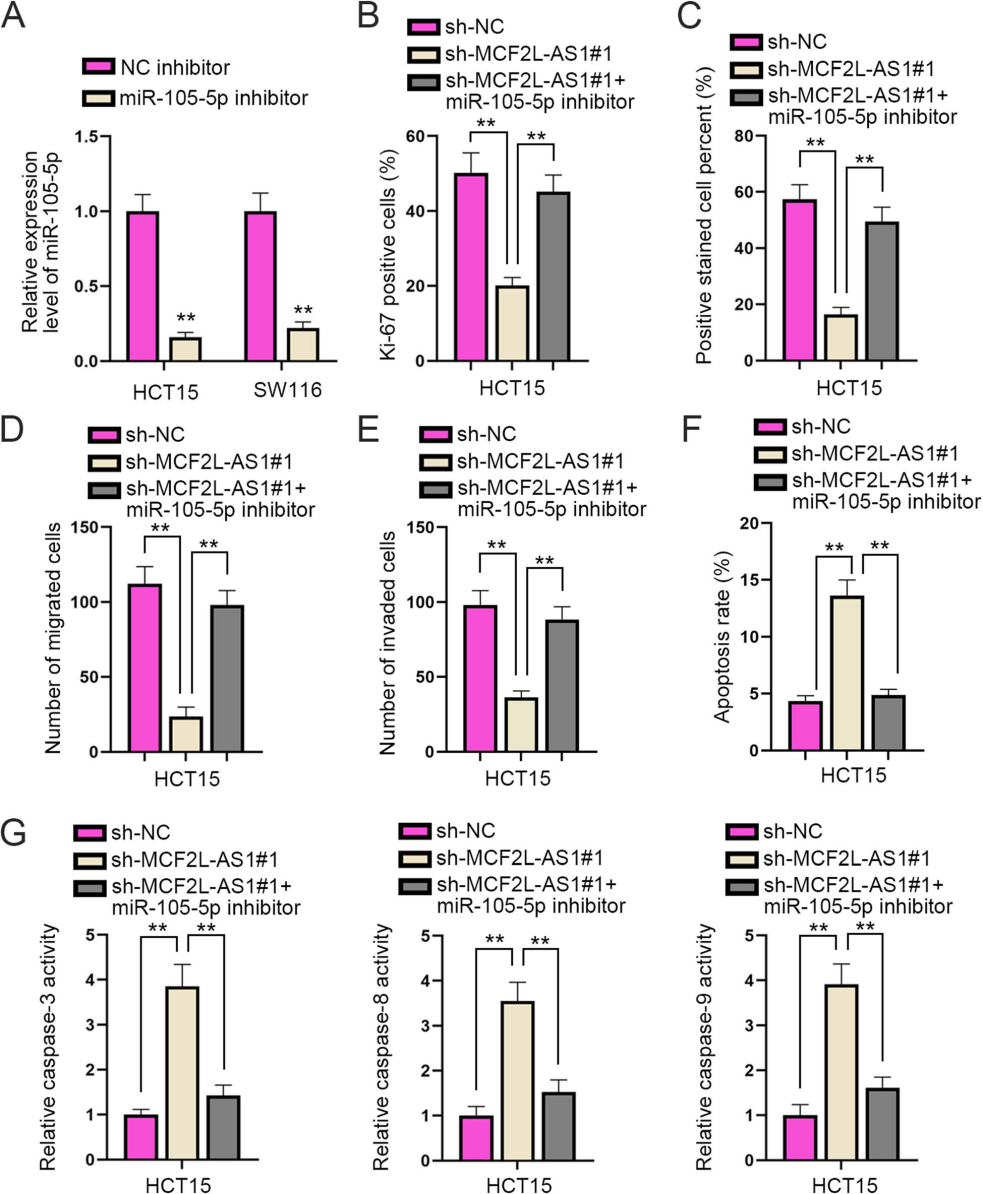
Knockdown of miR-105-5p reverses the effects of MCF2L-AS1 down-regulation on the progression of CRC. **A**. The interference efficiency of miR-105-5p was examined by RT-qPCR assay. **B** and **C**. Cell proliferation in different rescue groups was evaluated by IF and EdU assays. **D** and **E**. The migration and invasion of cells in different transfection groups were examined via Transwell assays. **F** and **G**. Cell apoptosis under different conditions was assessed by flow cytometry assay and detection of caspase-3/8/9 activities. The statistical analysis of Fig. 3A-G was tested with one-way ANOVA. **P < 0.01

Before we carried out a series of rescue assays to testify the interactive relationship between MCF2L-AS1 and miR-105-5p, HCT15 and SW116 cells were transfected with miR-105-5p inhibitor to silence miR-105-5p expression, and the interference efficiency of miR-105-5p was examined through RT-qPCR assay (Fig. 3**A**). Next, experimental groups were divided into groups including sh-NC, sh-MCF2L-AS1#1 and sh-MCF2L-AS1#1 + miR-105-5p inhibitor for the following rescue assays. According to the results of IF and EdU assays, the depressed CRC cell proliferation caused by sh-MCF2L-AS1#1 could be greatly reversed by the co-transfection with miR-105-5p inhibitor (Fig. 3B-C & Supplementary Fig. 1). As shown by the results of Transwell assays, MCF2L-AS1 silencing could apparently decrease the migratory and invasive abilities of CRC cell, but the co-transfection with miR-105-5p inhibitor could partially reverse the effect (Fig. 3D-E & Supplementary Fig. 1**)**. In addition, as observed from flow cytometry assay and caspase-3/8/9 activity analysis, miR-105-5p silencing could countervail the facilitating effect on the apoptosis of CRC cells caused by MCF2L-AS1 down-regulation (Fig. 3F-G & Supplementary Fig. 1). To conclude, knockdown of miR-105-5p countervails the inhibitory impact of MCF2L-AS1 silencing on the malignant development of CRC.

### RAB22A is a target of miR-105-5p and participates in CRC progression

**Fig. 4:**
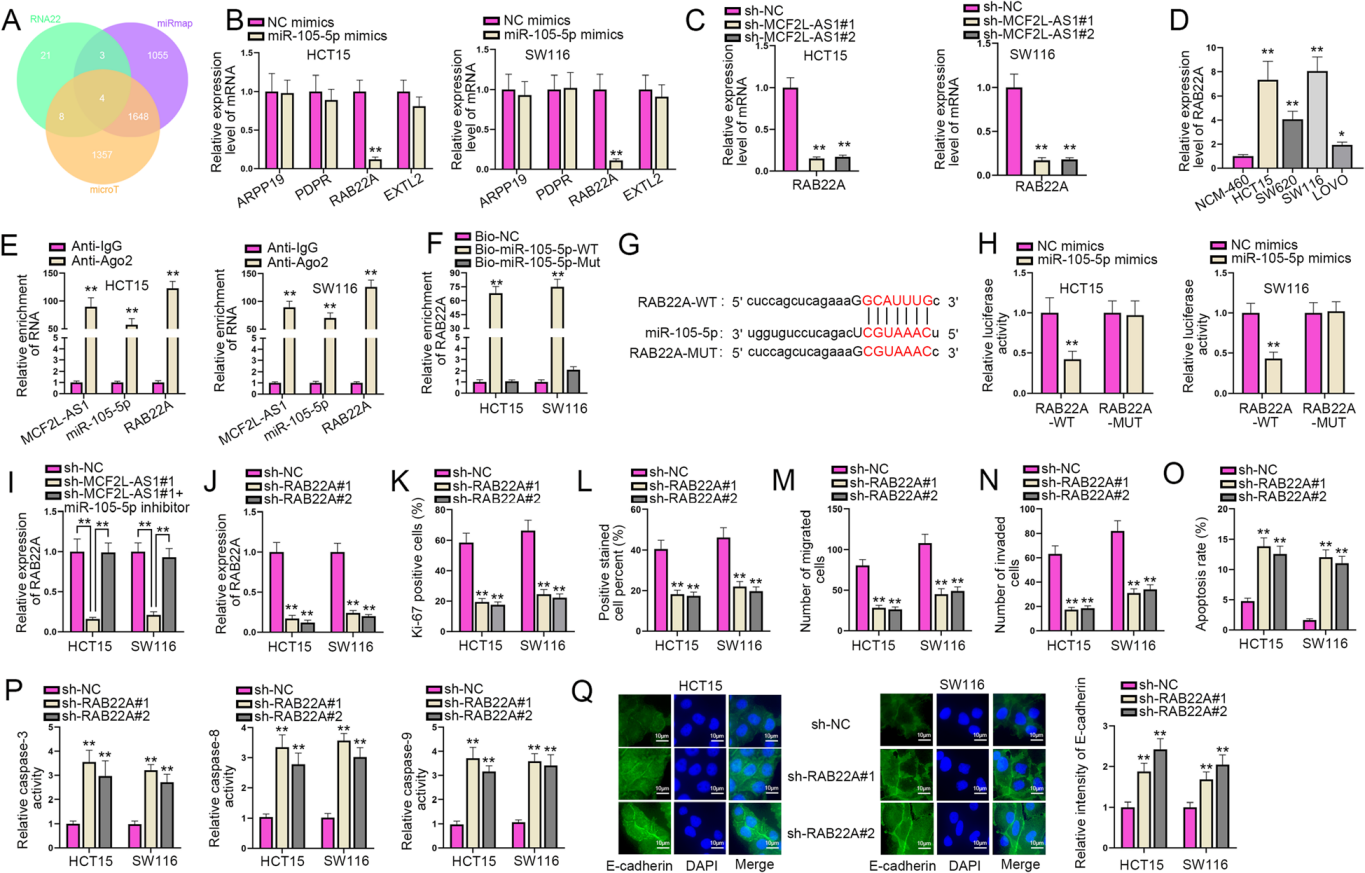
RAB22A is a target of miR-105-5p and participates in CRC progression. **A**. Bioinformatics tools (miRmap, microT and RNA22) were used to determine the potential target genes of miR-105-5p. **B**. The expression of candidate mRNAs was assessed via RT-qPCR analysis after up-regulating miR-105-5p. **C**. RAB22A expression was tested by RT-qPCR after down-regulating MCF2L-AS1. **D**. The expression of RAB22A was examined in CRC cell lines and human normal colon epithelial NCM-460 cell line. **E**. RIP assay was conducted to test the binding relationship among MCF2L-AS1, RAB22A and miR-105-5p. **F**. RNA pull down assay was carried out to test the connection between miR-105-5p and RAB22A. **G**. The binding sites between miR-105-5p and RAB22A were predicted by ENCORI website. **H**. The binding correlation between miR-105-5p and RAB22A was testified through luciferase reporter assay. **I**. The interplay among MCF2L-AS1, RAB22A and miR-105-5p was tested by RT-qPCR assay. **J**. The interference efficiency of RAB22A was tested via RT-qPCR assay. **K** and **L**. IF and EdU assays were adopted to assess the cell proliferation ability after RAB22A was silenced. **M** and **N**. Transwell assay was performed to evaluate cell migration and invasion upon RAB22A silencing. **O** and **P**. Flow cytometry assay and caspase-3/8/9 activity analysis were adopted to assess cell apoptosis in CRC under different transfection conditions when RAB22A was silenced. **Q**. The EMT process was detected through IF assay after RAB22A was knocked down in different groups. Adjustments of individual color channels were made on ‘Merge’ figures. The statistical analysis of Fig. 4H was tested with two-way ANOVA, and the statistical analysis of Fig. 4B-F, I-Q was tested with one-way ANOVA. *P < 0.05, **P < 0.01

As the significance of lncRNA-miRNA-mRNA mechanism in tumors has been manifested, it is necessary to identify the target gene of miR-105-5p [20]. Firstly, we utilized three bioinformatics tools, which are miRmap (https://mirmap.ezlab.org/), microT (http://www.microrna.gr/microT) and RNA22 (http://cbcsrv.watson.ibm.com/rna22.html) to predict the potential messenger RNAs (mRNAs) and 4 mRNAs were selected (ARPP19, PDPR, RAB22A and EXTL2) (Fig. 4A). RT-qPCR was then adopted to measure the expression of the above 4 mRNAs in HCT15 and SW116 cells under the transfection of miR-105-5p mimics, and the results showed that only RAB22A was significantly down-regulated by miR-105-5p mimics in HCT15 and SW116 cell lines (Fig. 4B). Next, we used RT-qPCR assay to further verify the expression of RAB22A upon MCF2L-AS1 silencing, and it was shown that RAB22A expression was declined by upon MCF2L-AS1 silencing in HCT15 and SW116 cells (Fig. 4C). After that, the expression level of RAB22A in CRC cells was tested via RT-qPCR, and results uncovered that RAB22A was highly expressed in CRC cell lines than that in human normal colon epithelial NCM-460 cells (Fig. 4D). To continuously verify whether RAB22A was the target downstream gene of miR-105-5p, RIP and RNA pull down assays were conducted respectively. As showed in Fig. 4E & Supplementary Fig. 2A, MCF2L-AS1, miR-105-5p and RAB22A were enriched in Anti-Ago2 group rather than in Anti-IgG group, indirectly implying that those three RNAs could co-exist in RNA-induced-silencing-complex (RISC). Meanwhile, it was shown that the enrichment of RAB22A was elevated in the wild type of biotinylated miR-105-5p (Bio-miR-105-5p-WT) compared to the mutant type of biotinylated miR-105-5p (Bio-miR-105-5p-Wut) (Fig. 4F). The binding sites between miR-105-5p and RAB22A were predicted via ENCORI website and presented in Fig. 4G. After that, luciferase reporter assay was adopted to measure the luciferase activity change under different conditions. As shown by the results, miR-105-5p overexpression could effectively cut down the luciferase activity of wild type of RAB22A (RAB22A-WT) but not the mutant type of RAB22A (RAB22A-Mut) in CRC cells compared to NC mimics (Fig. 4H), suggesting the binding ability between RAB22A and miR-105-5p. Moreover, it was shown by rescue assays that RAB22A expression was notably reduced upon MCF2L-AS1 silencing while this result could be greatly rescued by miR-105-5p down-regulation (Fig. 4I). Therefore, it is concluded that MCF2L-AS1/miR-105-5p/RAB22A axis could act as a regulatory network in CRC.

As for RAB22A, we also detected its functions in CRC cells. First of all, sh-RAB22A#1 and sh-RAB22A#2 was transfected into CRC cells and RT-qPCR assay was then utilized to examine the interference efficiency of RAB22A in CRC cells (Fig. 4J). Subsequently, IF staining and EdU assays were adopted to assess the cell proliferation of CRC cells upon RAB22A silencing, and the results showed that both the Ki-67 positive cells and EdU positive cells were reduced by down-regulation of RAB22A, indicating that RAB22A knockdown could effectively suppress CRC cell proliferation (Fig. 4K-L & Supplementary Fig. 2B-C). After that, results of Transwell assay reflected that RAB22A silencing could significantly weaken cell migration and invasion of CRC cells (Fig. 4M-N & Supplementary Fig. 2D-E). In addition, flow cytometry assay and caspase-3/8/9 activity analysis were adopted to testify the impact of RAB22A inhibition on cell apoptosis, and it was shown that the apoptosis rate of HCT15 and SW116 cells and the activity of caspase-3/8/9 were enhanced upon RAB22A silencing, which showed that RAB22A knockdown could effectively enhance CRC cell apoptosis (Fig. 4O-P and Supplementary Fig. 2F). Besides, it was shown from IF assay that the down-regulation of RAB22A could lead to the up-regulation of E-cadherin level, indicating that RAB22A silencing could repress the EMT process of CRC cells (Fig. 4Q). Taken together, RAB22A is the target of miR-105-5p and aggravates CRC progression.

### RAB22A overexpression attenuates the inhibitory effects of MCF2L-AS1 silencing on CRC progression

**Fig. 5:**
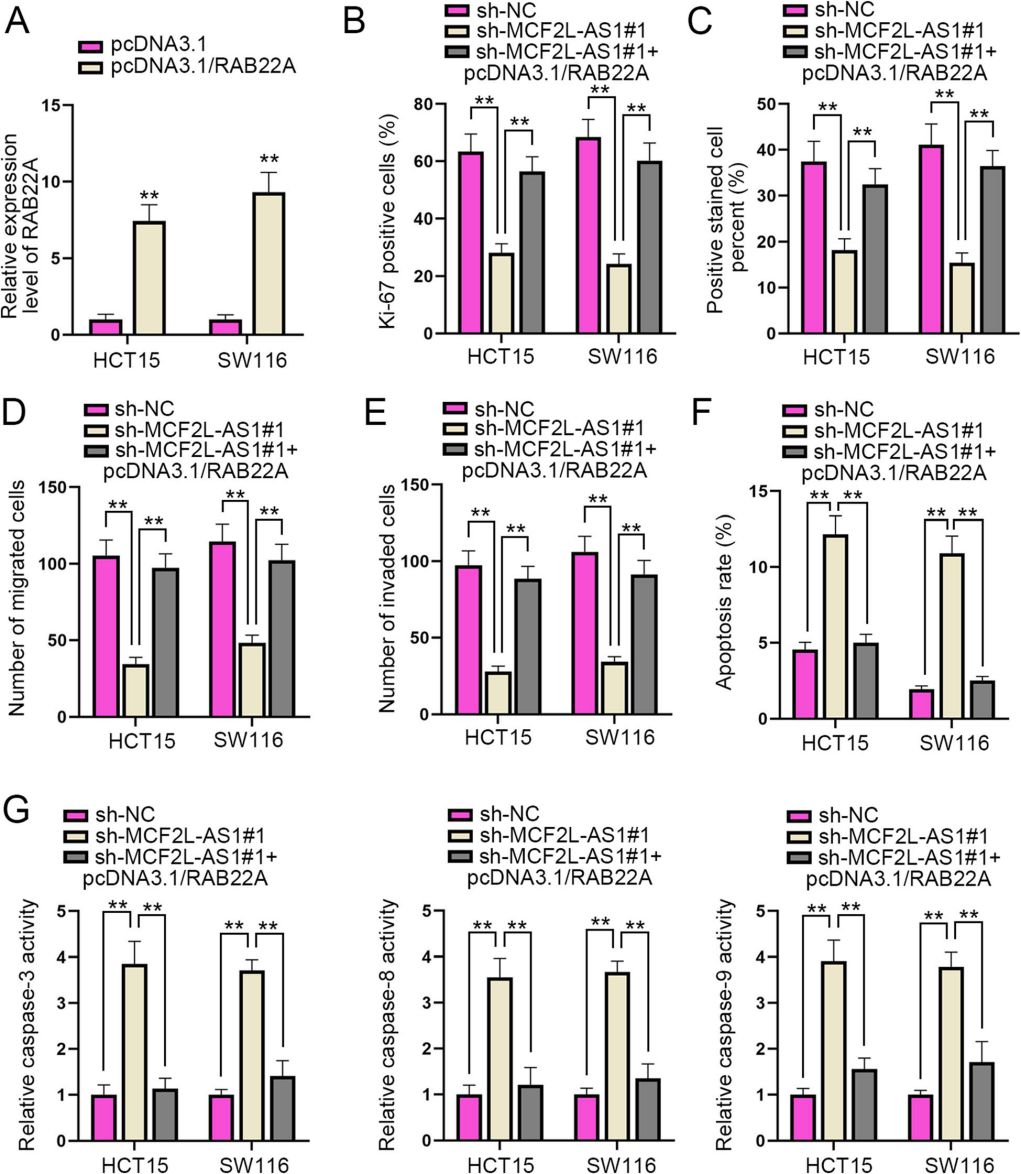
RAB22A neutralizes the suppressive effects of MCF2L-AS1 on CRC progression. **A**. The overexpression efficiency of RAB22A was examined by RT-qPCR assay. **B** and **C**. Cell proliferation in different rescue groups was evaluated by IF and EdU assays. **D** and **E**. The migration and invasion of cells in different groups were examined via Transwell assay. **F** and **G**. Cell apoptosis in different groups was assessed by flow cytometry assay and caspase-3/8/9 activity analysis. The statistical analysis of Fig. 5A-G was tested with one-way ANOVA. **P < 0.01

In the last part, a series of rescue experiments were conducted to verify how the MCF2L-AS1 and RAB22A affected the malignant development of CRC cells. The overexpression efficiency of RAB22A was firstly examined via RT-qPCR (Fig. 5A). Experimental groups for rescue assays were then divided into sh-NC, sh-MCF2L-AS1#1 and sh-MCF2L-AS1#1 + pcDNA3.1/RAB22A groups. IF staining as well as EdU assays was utilized to assess cell proliferation in the above transfection groups, and the results showed that decreased MCF2L-AS1 expression could weaken cell proliferation, whereas this effect could be partially reversed by the co-transfection with pcDNA3.1/RAB22A (Fig. 5B-C & Supplementary Fig. 1). Next, Transwell assays were adopted to validate cell migration and invasion under different transfection groups. Results showed that the repressed cell migration and invasion mediated by sh-MCF2L-AS1#1 was significantly reversed by the co-transfection with pcDNA3.1/RAB22A (Fig. 5D-E & Supplementary Fig. 1). In the end, flow cytometry assay and caspase-3/8/9 activity analysis were conducted to examine CRC cell apoptosis in different groups. As shown by the results, it was suggested that the enhanced cell apoptosis induced by MCF2L-AS1 silencing could be recovered by the up-regulation of RAB22A (Fig. 5F-G). In conclusion, RAB22A overexpression can reverse the inhibitory effect on CRC progression caused by MCF2L-AS1 silencing.

## Discussion

LncRNAs, as important biological makers for human diseases or cancers [21–23], have been extensively studied in the past few years. The aberrant expression of lncRNAs has been demonstrated to exert important influence on the biological behaviors of cells. MCF2L-AS1 serves as a relatively newly found lncRNA and its role in various tumors remains unclear. When we search for related studies, we found that MCF2L-AS1 was verified to be involved in CRC [14, 15] and could promote CRC progression. Therefore, we chose MCF2L-AS1 as the study object with the purpose of exploring the underlying mechanism of MCF2L-AS1 in CRC development and comparing our findings with those disclosed by the previous researches. Accordingly, in our study, MCF2L-AS1 was discovered to be highly-expressed in CRC calls and it could accelerate CRC cell proliferation, migration, invasion, EMT process while inducing cell apoptosis, which was consistent with the previous findings.

CeRNA pattern is one of the main mechanisms of post-transcriptional regulation [24], in which lncRNAs possibly play an important part to regulate their target genes. In this study, according to the results of subcellular fractionation and FISH assays, it was demonstrated that MCF2L-AS1 was mainly distributed in the cytoplasm of CRC cells, which indicated the potential ceRNA pattern. Therefore, we further explored the potential target of MCF2L-AS1, and miR-105-5p was selected and verified as the target micRNA that could bind to MCF2L-AS1. Furthermore, a series of functional assays were conducted and we proved that miR-105-5p was with low expression in CRC cells with anti-proliferative, anti-migratory and pro-apoptotic features. Many previous reports have revealed the role of miR-105-5p. For example, CD44 sponges miR-105-5p to regulate PES1 in liver cancer stem cells to push tumor growth [25]. In the Idiopathic Parkinson’s disease, miR-105-5p is considered as underlying effective biomarker [26]. In addition, the present study unveiled that miR-105-5p could be treated as a potential biomarker for cancer treatment.

Aside from lncRNAs and miRNAs, mRNAs can exert important functions by building the pathway with lncRNAs and miRNAs in different tumors or diseases [20, 27, 28]. For example, lnc-RI acts as a ceRNA to stabilize RAD51 mRNA via competitively combining with miR-193a-3p [29]. HOTAIR modulates CCND1 and CCND2 expression through regulating miR-206 expression in ovarian cancer [30]. Here, RAB22A was found and verified to be the target gene of miR-105-5p, and thus the MCF2L-AS1/miR-105-5p/RAB22A axis was built up in CRC cells. Furthermore, the effects of RAB22A mRNA in CRC was explored in detail and the results presented that RAB22A silencing could inhibit CRC progression. According to previous studies, RAB22A has been uncovered, as a member of oncogene family, to promote melanoma growth and renal cell carcinoma development [31, 32]. Those findings, together with the study outcomes in our study, all demonstrated the oncogenic function of RAB22A in cancer development, which indicated its therapeutic value for cancer treatment.

## Conclusion

Our study indicated that MCF2L-AS1 could accelerate cell proliferation, migration, invasion and EMT progression, while reduced cell apoptosis via regulating the miR-105-5p/RAB22A axis in CRC, which suggested that MCF2L-AS1 could be treated as a novel biomarker as well as a therapeutic target for CRC treatment in the future.

## Supplementary Information

**Additional file 1 Supplementary Fig. 1** Supporting figures of results obtained under microscopes were presented. Adjustments of individual color channels were made on ‘Merge’ figures.

**Additional file 2 Supplementary Fig. 2 A**. RIP assay was conducted to test the binding relationship among MCF2L-AS1, RAB22A and miR-105-5p. **B** and **C**. IF staining and EdU assay was adopted to evaluate the cell proliferation ability of cells upon RAB22A silencing. **D-E**. Transwell assay was utilized to measure cell migration and invasion after RAB22A was knocked down. **F**. Flow cytometry analysis was adopted to assess cell apoptosis of CRC cells when RAB22A was knocked down. The above figures are the experimental images of Fig. 4E, K, L, M, N and O. Adjustments of individual color channels were made on ‘Merge’ figures.

**Additional file 3 Supplementary File 1** Original results of western blot assay were presented.

**Additional file 4 Supplementary Table 1** Related information on plasmids quantities and transfection concentration was presented.

## Data Availability

The datasets generated and/or analysed during the current study are not publicly available but are available from the corresponding author on reasonable request.

## Abbreviations

MCF2L-AS1: MCF.2 cell line derived transforming sequence like antisense RNA1
RAB22A: Ras-related protein Rab-22A
lncRNAs: Long non-coding RNAs
miRNAs: MicroRNAs
mRNAs: Messenger RNAs
ceRNA: Competing endogenous RNA
ATCC: American Type Culture Collection
FBS: Fatal Bovine Serum
NC: Negative Control
qPCR: Quantitative reverse transcription real-time polymerase chain reactionRT-
EdU: 5-Ethynyl-2′-deoxyuridine
DAPI: 4′,6-diamidino-2-phenylindole
FISH: Fluorescence in situ hybridization
RIP: RNA immunoprecipitation
RISC: RNA-induced-silencing-complex
WT: Wild-type
Mut: Mutant
SD: Standard deviation
ANOVA: Analysis of Variance
