# Supplementary figures and images for: LncRNA MCF2L-AS1 aggravates the malignant development of colorectal cancer via targeting miR-105-5p/RAB22A axis
Source: BMC Cancer. 2021 Sep 30;21:1069. doi: 10.1186/s12885-021-08668-w (PMC8482615; doi:10.1186/s12885-021-08668-w)

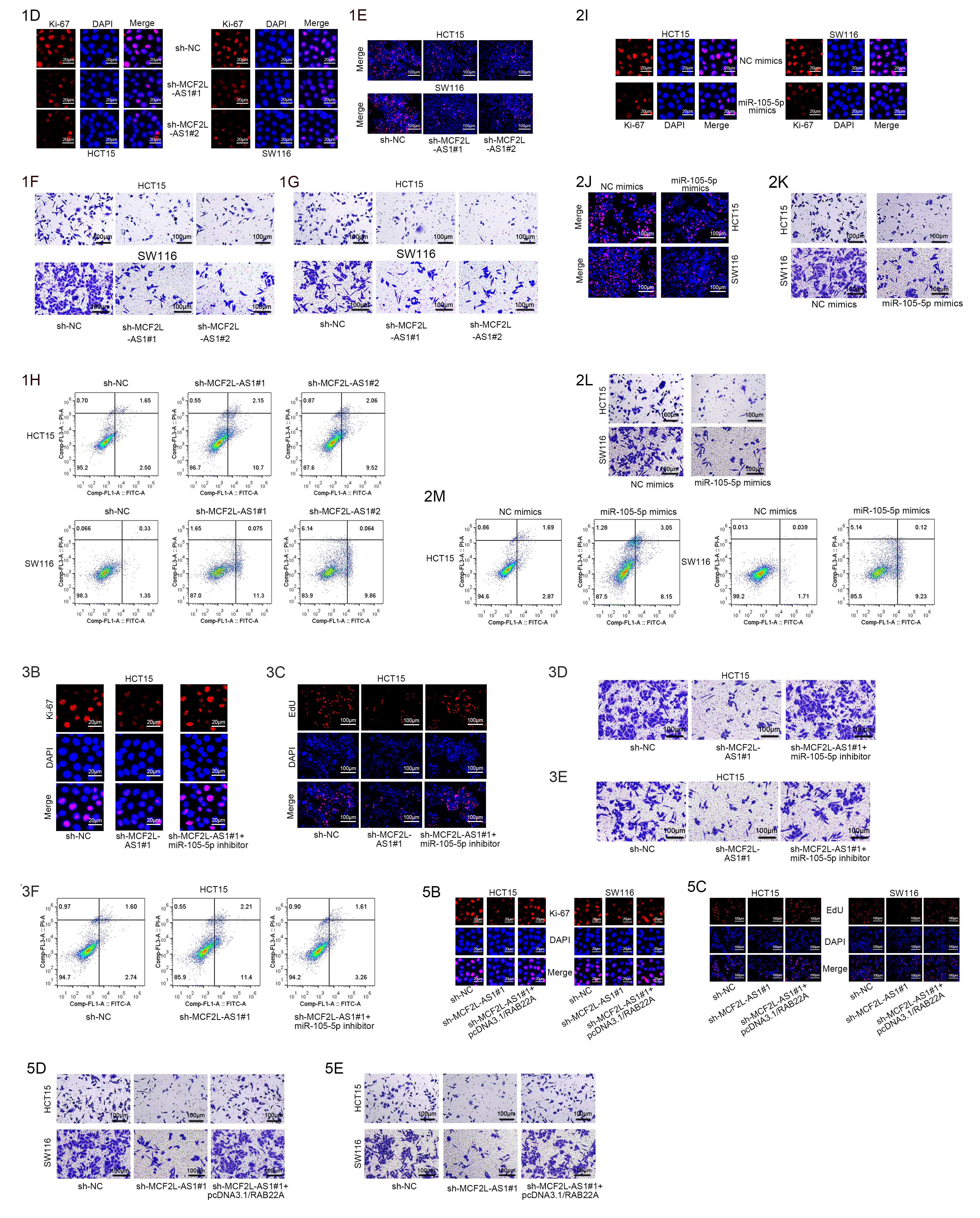

Supplement: Supplementary file 1 — Additional file 1 Supplementary Fig. 1 Supporting figures of results obtained under microscopes were presented. Adjustments of individual color channels were made on ‘Merge’ figures. [file 12885_2021_8668_MOESM1_ESM.tif]

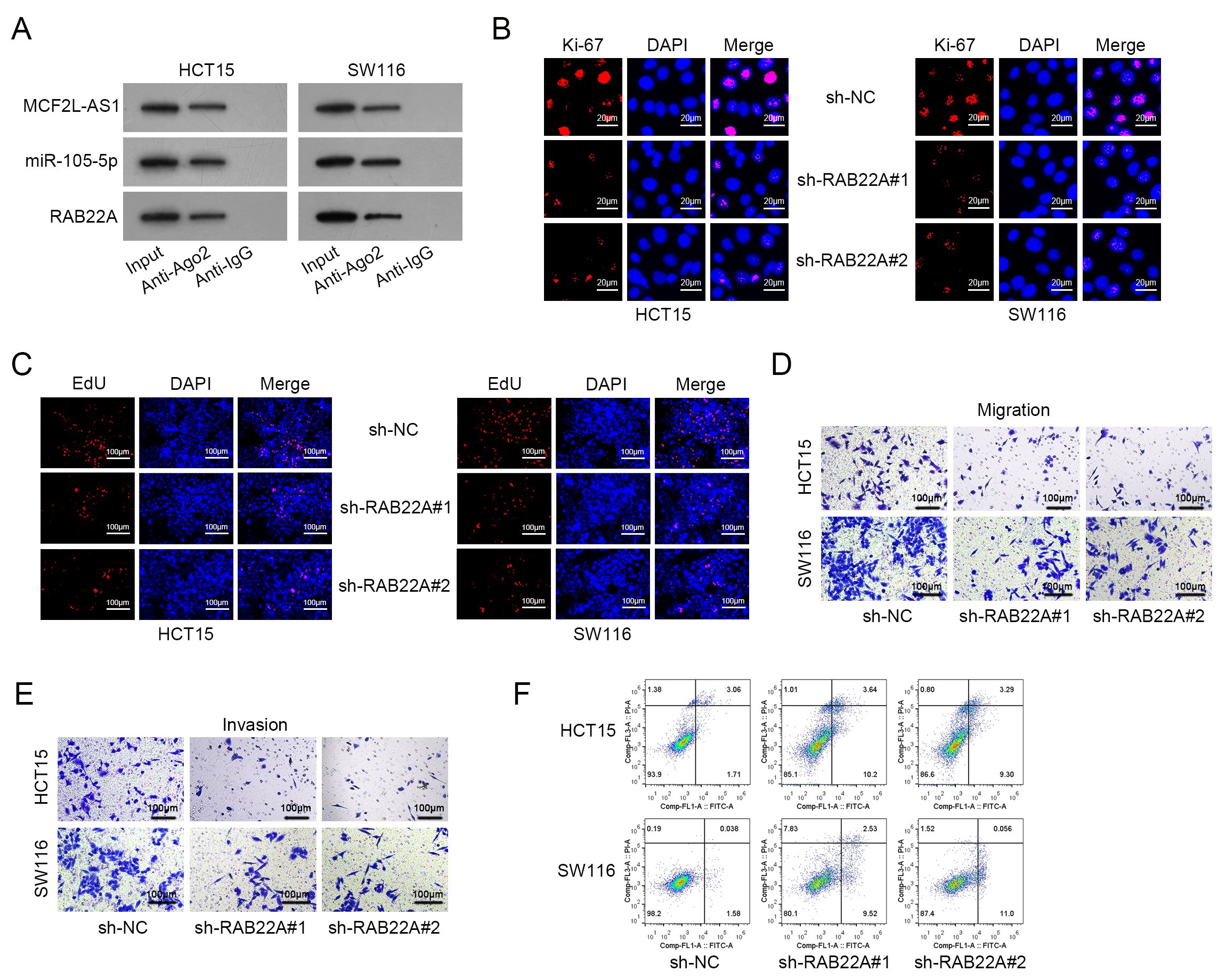

Supplement: Supplementary file 2 — Additional file 2 Supplementary Fig. 2 A. RIP assay was conducted to test the binding relationship among MCF2L-AS1, RAB22A and miR-105-5p. B and C. IF staining and EdU assay was adopted to evaluate the cell proliferation ability of cells upon RAB22A silencing. D-E. Transwell assay was utilized to measure cell migration and invasion after RAB22A was knocked down. F. Flow cytometry analysis was adopted to assess cell apoptosis of CRC cells when RAB22A was knocked down. The above figures are the experimental images of Fig. 4E, K, L, M, N and O. Adjustments of individual color channels were made on ‘Merge’ figures. [file 12885_2021_8668_MOESM2_ESM.tif]

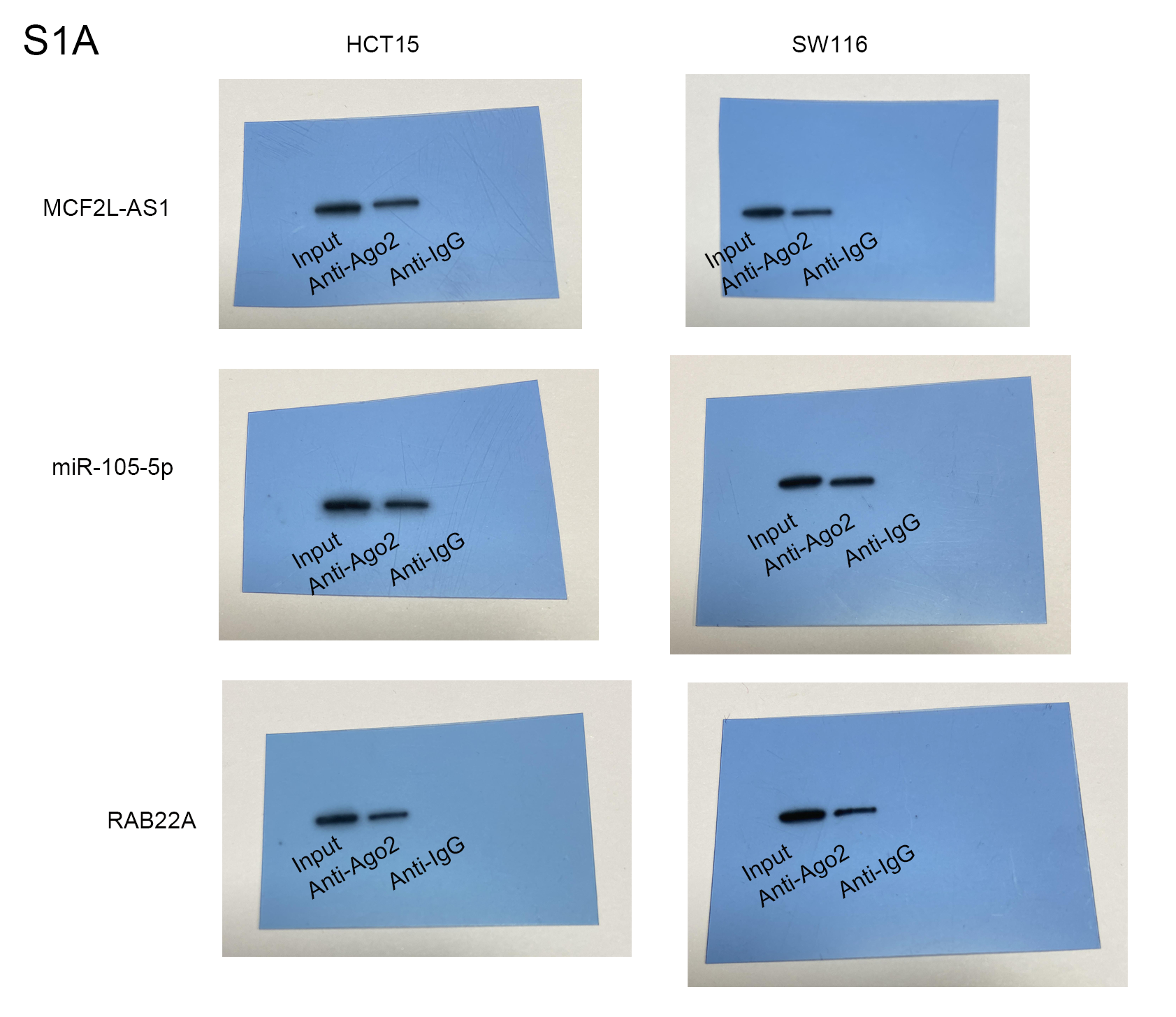

Supplement: Supplementary file 3 — Additional file 3 Supplementary File 1 Original results of western blot assay were presented. [file 12885_2021_8668_MOESM3_ESM.tif]
